# Is medical education hazardous to your health?

**Published:** 2014-12-17

**Authors:** Marcel D’Eon

**Affiliations:** University of Saskatchewan, Saskatoon, Saskatchewan, Canada

When I entered “medical student stress” into the search window for Google Scholar I got over 1.5M hits. Judging by my own experience and the few (compared to 1.5M) articles I have read, we don’t find a very happy family portrait. Many of the articles which compose this issue of CMEJ speak to high levels of stress and burnout, worries about finding residency positions and jobs, and the experience of various forms of harassment. Medical education can sometimes be harsh rather than happy. Sorry; we are only the messengers here. Rather than drawing weapons and flailing them at the screen in front of you, let’s deal with this situation as best we can. I believe the first step is acknowledging the nature and magnitude of the problem, then recommending and implementing usually short term treatments, and finally – but perhaps most importantly – moving upstream^1^ to address the sources of stress.

The literature is replete with studies that identify the rather serious consequences of stress: depression,^2^ decreased job satisfaction and disillusionment with the medical profession,^2^,^3^ psychological distress,^2^,^4^ absenteeism and disability,^2^ exhaustion and decreased motivation.^3^ This is clearly an important issue.

To address these problems, some schools have wellness programs of various kinds. There are extracurricular stress reduction programs actually implemented^2^,^5^,^6^ or recommended.^7^ I am quite sure the vast majority of Canadian and US medical schools have and make readily available various forms of support and counselling to their trainees as strongly suggested by Benbassat et al.^7^ These measures are a good start.

It seems few places have successfully addressed the sources of some of these stressors. As reported by Slavin, Schindler and Chan, Saint Louis University School of Medicine made changes to course content, contact hours, scheduling, grading, and electives adding learning communities and resilience/mindfulness experiences for all students beginning in 2009–2010.^8^ Compared to previous years, these changes were associated with significantly lower levels of depression and anxiety symptoms and stress, as well as significantly higher levels of community cohesion. Though we may have the Hawthorne Effect here,^9^ Slavin et al. bring very promising news. Benbassat et al. similarly addressed the source of one of the stressors: the incongruence between the heavy bio-medical content especially in the first two years of medical training, and the realities of clinical practice where a broader biopsychosocial model with a greater emphasis on upstream prevention seems more appropriate.^7^ This disconnect then leaves medical students without the knowledge and skills needed to be effective clinicians while at the same time wondering about the futility of memorizing uncounted minutiae of microbiology and anatomy. Benbassat et al. recommended that medical schools close this gap as a strategy to reduce stress, though they did not test this empirically.^7^ Undoubtedly there are other promising ideas out there that have not yet been tested and some others like the Saint Louis University School of Medicine that could (and should) be replicated and studied in greater depth.

One might balk at curricular strategies and solutions to reduce stress given concerns about the quality of the MD program and the extensive amount and complexity of skills and knowledge needed for residency. However, curricular reform to reduce stress is a win-win proposition. An overcrowded curriculum creates many learning pathologies.^10^–^14^ By rejecting filter failure^15^ on our part and instead embracing and implementing proper content management practices,^16^ we may not only be able to reduce stress (which in and of itself would help improve learning) but we may also simultaneously facilitate long term and deep learning as well as enhanced self-directed learning for our students.^17^ Considering the potential to reduce medical student stress and improve learning outcomes, curricular reform seems to be a compelling option.

In this issue of the CMEJ we are very pleased to publish several studies of special interest to Canadian medical educators, with implications for medical education across the globe. From British Columbia to the Rock, authors and editors from across Canada have made this issue possible. We cover stress and resilience, education opportunities, and clinical skills training for medical students as well as research, intimidation and harassment, burnout, and job prospects for residents.

Rahimi et al., using a cross-sectional design, documented the extent of medical student stress, coping, and resilience at a Canadian medical school. They found higher stress, more negative coping, and lower resilience than age and gender-matched peers in the general population and suggest both greater self-care for medical trainees and curricular change.

Rutherford and Oda set out using focus groups to understand how burnout is perceived and experienced by family medicine residents at five different sites associated with a different Canadian University, and to identify both contributory and protective factors. About 70% of the focus group participants met criteria for burnout described by such phenomenon as physical and emotional exhaustion, isolation from loved ones, and disillusionment with the medical profession. Contributory factors included high workload, burned-out colleagues, and perceived undervaluing of family medicine. Protective factors included strong role models in medicine, feeling that one’s work is valued, and rotations in family medicine. They recommended reducing the contributory factors and strengthening the protective factors. Now the hard work begins!

Hallet et al. examined another source of considerable stress well beyond the power of the medical school to address alone: jobs. Using surveys of residents, they set out to analyze physicians’ employment issues in Quebec and their impact on residents’ training in specialty programs. Their study revealed that 47.3% of graduates did not have a position two months before finishing their training. Among the 47.3% of residents without a position, 27.1% of them intended to leave Quebec, and 19.6% to complete a fellowship to postpone their start in practice. Overall, 77.9% of all respondents believed there are not enough job opportunities for the number of trainees. Imagine what it must be like after four years of medical school (with several years of university training before that) with two to five years of residency training and you are not sure that there will be a job waiting for you. For many of our readers, this is your life.

Karras et al. described second and third year medical students’ perceptions of and attitudes toward working with children before and after pediatric clinical skills teaching sessions, and the experiences of students taught by pediatricians only compared to students working with a combination of pediatricians and family physicians. This is particularly relevant given the recommendation around generalism in the Future of Medical Education in Canada report.^18^ Students in two classes were surveyed before and after small group clinical skills instructional sessions and post instructional reflections were analyzed. While they found that students already had positive attitudes toward the medical care of children, they also found that students who had been taught by family physicians identified prevention, health promotion and multidisciplinary care in their reflections.

In Karim and Duchcherer, we encounter a literature review of international scope to determine the prevalence, key themes and solutions to intimidation and harassment across residency programs. Intimidation and harassment continue to be prevalent with 45–93% of residents reporting that they experienced intimidation and harassment on at least one occasion. Verbal abuse was the most common form with staff physicians and nurses the dominant source. The authors affirm that very few incidents of inappropriate behaviour were reported to residency programs, though this intimidation and harassment caused significant emotional impact. They recommend that the issues of intimidation and harassment be revisited, solutions sought, plans made, and programs implemented.

O’Brien and D’Eon questioned why we insist that residents engage in original clinical research. Taking into account both social accountability and public welfare, they argue for considering the opportunity costs of mandatory original clinical research by residents and encourage research training aimed at the public good in the local setting.

Evren et al. provided a brief overview of osteopathic medical education and training in the United States. Why? Because Doctors of osteopathic medicine programs provide an additional pathway for Canadian students interested in pursuing a medical education, as well as potential for a successful post-graduate US residency match.

In this issue you will find some studies that might cause you to reflect more deeply on medical education, become angry, or resolve to help change the system. Hopefully all three!
